# Supplementary material for: Dental Pulp Stem Cells Modulate Inflammasome Pathway and Collagen Deposition of Dermal Fibroblasts
Source: Cells. 2024 May 14;13(10):836. doi: 10.3390/cells13100836 (PMC11120068; doi:10.3390/cells13100836)
Supplement: Supplementary file 1 [file cells-13-00836-s001.zip › cells-2914504-supplementary.pdf]

cells

MDPI

Supplementary Materials

A.

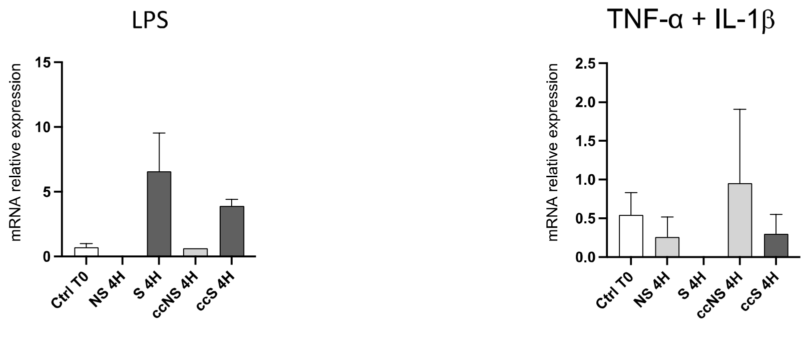

B.

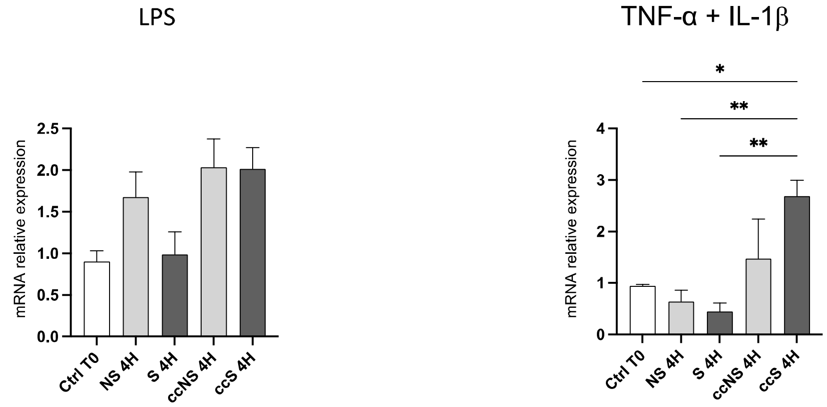

C.

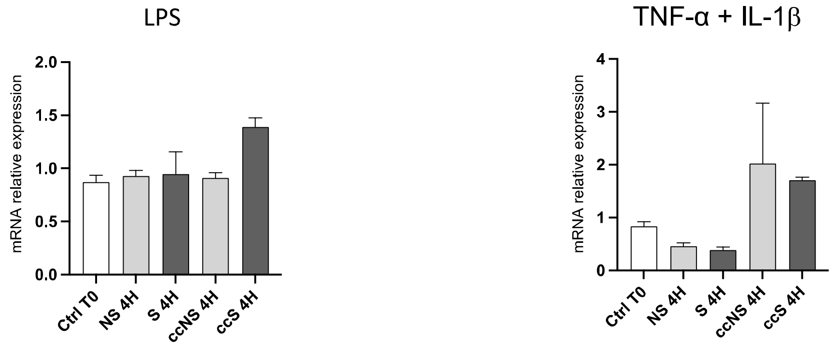

Supplementary Figure S1. A. Relative expression of the gene encoding NLRP3 in fibroblasts treated with LPS (left panel) or TNF-*α* and IL-1β (right panel) for four 4 h, alone or in co-culture with DPSCs. Data are reported as fold change respect to Ctrl T0, set to 1, and shown as mean ± SD of three independent experiments. **B.** Relative expression of the genes encoding NAIP in fibroblasts treated with LPS (left panel) or TNF-*α* and IL-1β (right panel) for four 4 h, alone or in co-culture with DPSCs. Data are reported as fold change respect to Ctrl T0, set to 1, and shown as mean ± SD of three independent experiments. **C.** Relative expression of the genes encoding PYCARD in fibroblasts treated with LPS (left panel) or TNF-*α* and IL-1β (right panel) for four 4 h, alone or in co-culture with DPSCs. Data are reported as fold change respect to Ctrl T0, set to 1, and shown as mean ± SD of three independent experiments. Ctrl: fibroblasts before stimulation, without co-culture; NS: non-stimulated; S: stimulated; ccNS: non-stimulated in co-culture with DPSCs; ccS: stimulated in co-culture with DPSCs. *=p<0.05; **=p<0.01.

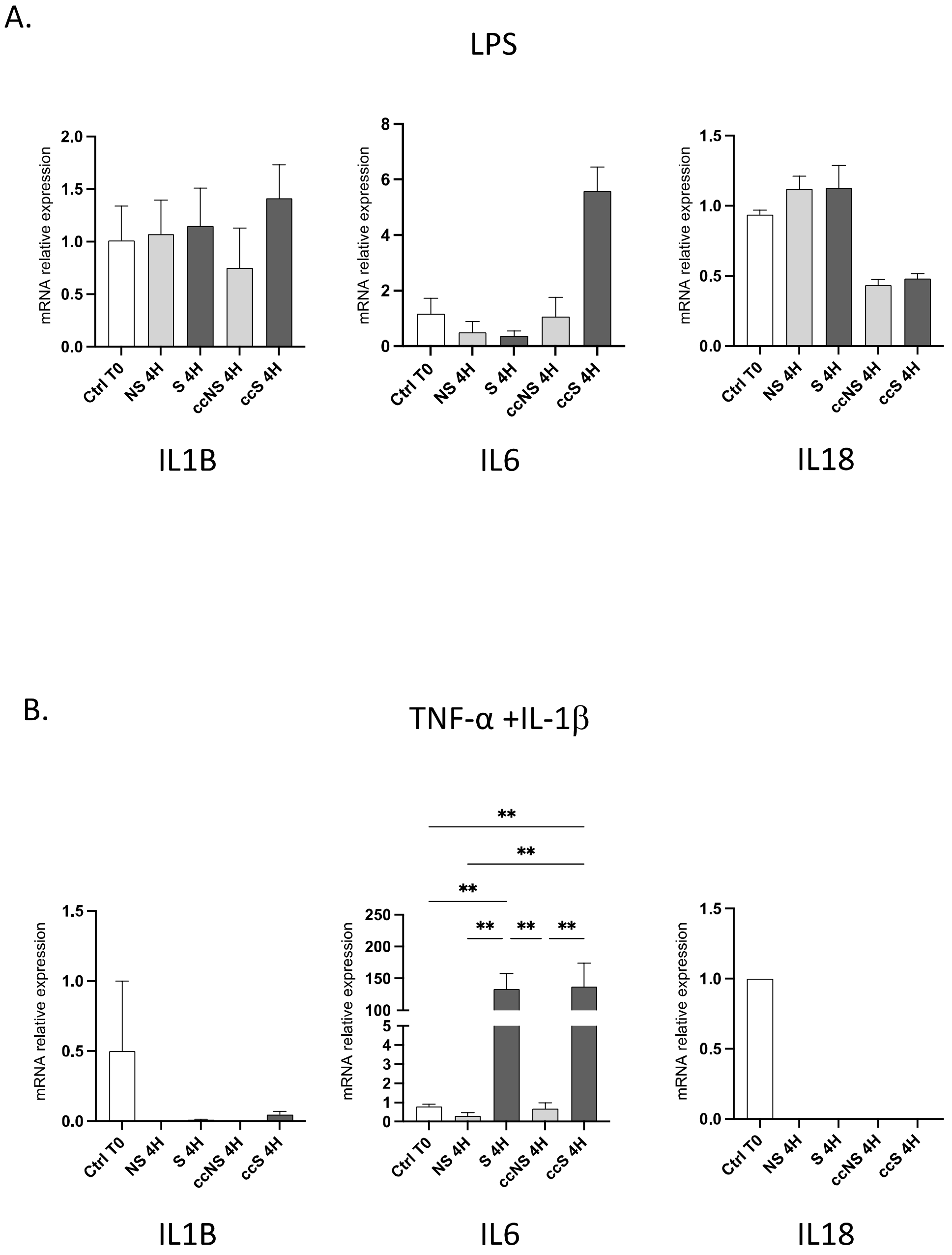

Supplementary Figure S2. A. Relative expression of the genes encoding pro-inflammatory cytokines IL-1β, IL-6, and IL-18 in DPSCs treated with LPS (upper panels) or TNF-*α* and IL-1β (lower panels) for four 4 h, alone or in co-culture with fibroblasts. Data are reported as fold change respect to Ctrl T0, set to 1, and shown as mean ± SD of three independent experiments. **B.** Relative expression of the genes encoding pro-inflammatory cytokines IL-1β, IL-6 and IL-18 in DPSCs treated with TNF-*α* and IL-1β for four 4 h, alone or in co-culture with fibroblasts. Data are reported as fold change respect to Ctrl T0, set to 1, and shown as mean ± SD of three independent experiments. Ctrl: DPSCs before stimulation, without co-culture; NS: non-stimulated; S: stimulated; ccNS: non-stimulated in co-culture with fibroblasts; ccS: stimulated in co-culture with DPSCs. *=p<0.05; **=p<0.01; ***=p<0.001; ****=p<0.0001.

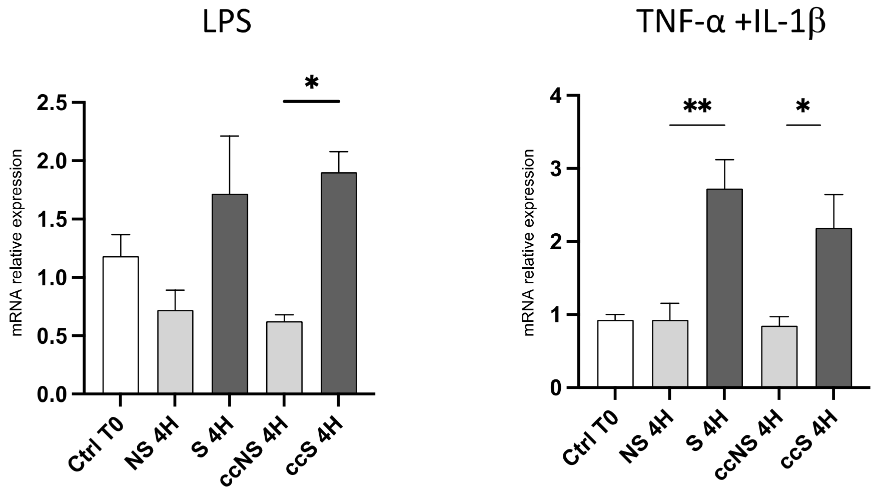

Supplementary Figure S3. Relative expression of the gene encoding TRL4 in fibroblasts treated with LPS (left panel) or TNF-α and IL-1β (right panel) for four 4 h, alone or in co-culture with DPSCs. Data are reported as fold change respect to Ctrl T0, set to 1, and shown as mean ± SD of three independent experiments. Ctrl: fibroblasts before stimulation, without co-culture; NS: non-stimulated; S: stimulated; ccNS: non-stimulated in co-culture with DPSCs; ccS: stimulated in co-culture with DPSCs. *=$p<0.05$; **=$p<0.01$.

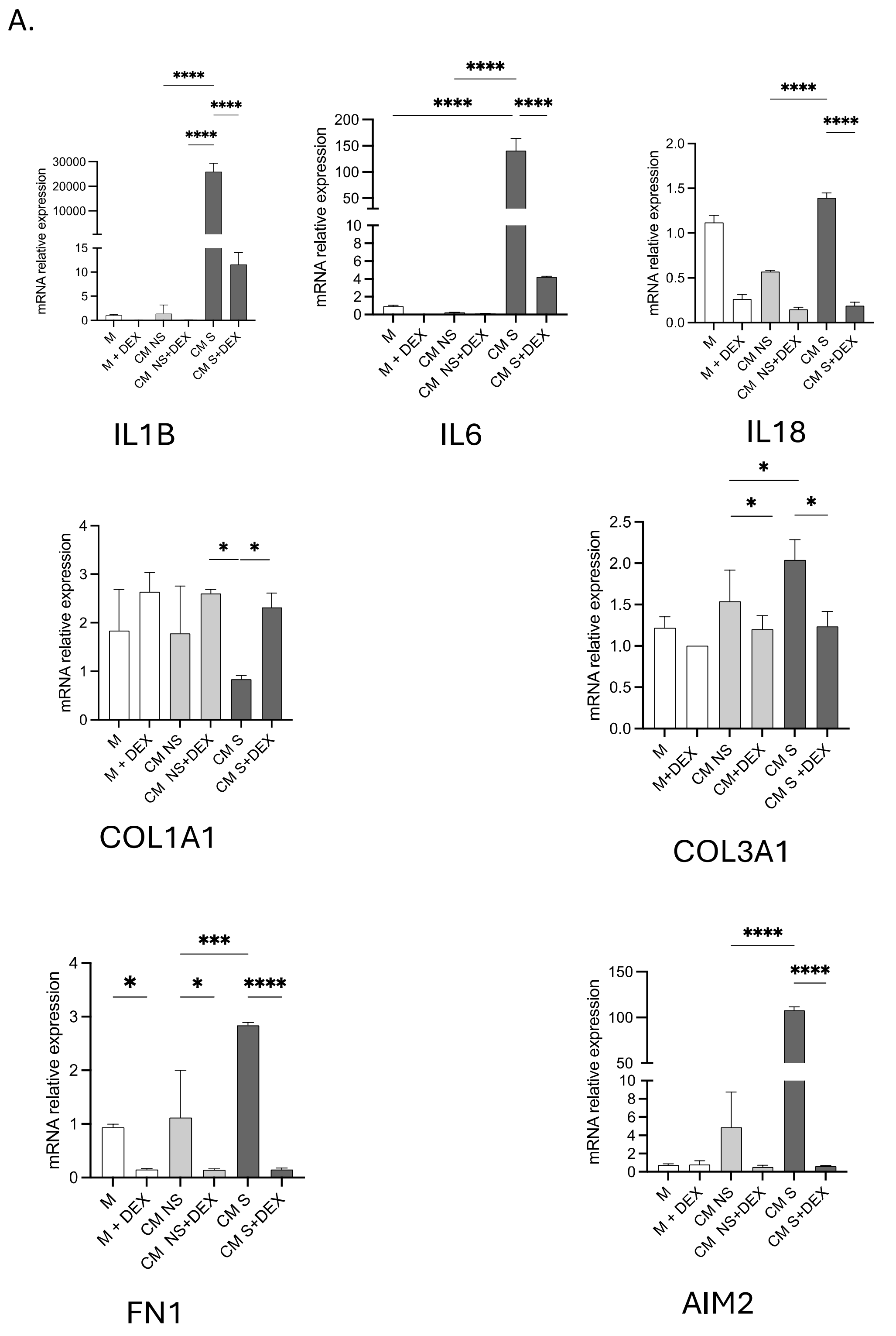
A.
mRNA relative expression
M
M + DEX
CM NS
CM NS+DEX
CM S
CM S+DEX
****
*
***
IL1B
IL6
IL18
COL1A1
M+DEX
CM+DEX
CM S +DEX
COL3A1
FN1
AIM2

B.

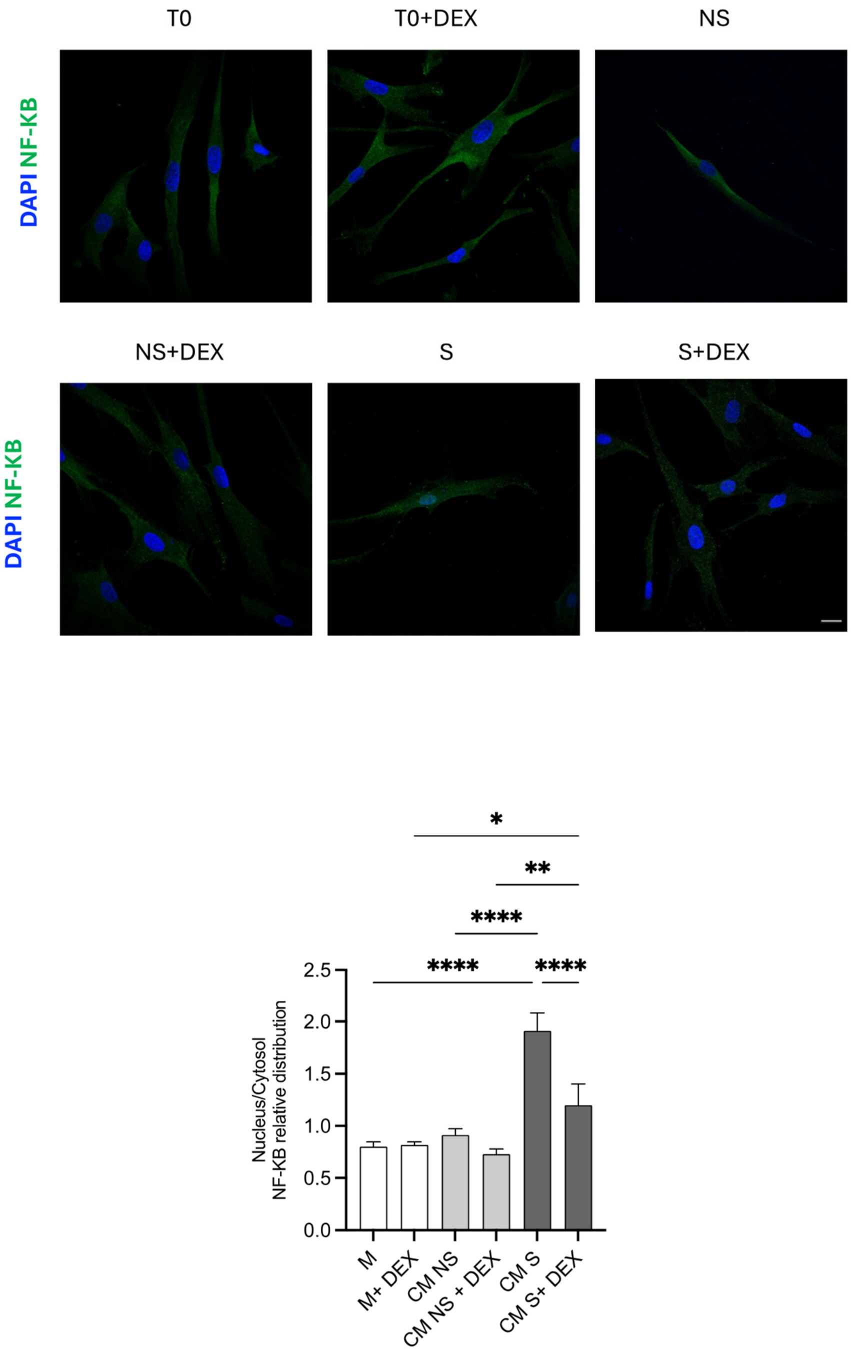

Supplementary Figure S4. A. Relative expression of the genes encoding IL1B, IL6, IL18, COL1A1, COL3A1, FN1 and AIM2 in fibroblasts cultured for 4 hours in a medium conditioned by DPSCs, in the presence or absence of Dexamethasone (DEX) 1 μM. Data are reported as fold change respect to Ctrl T0, set to 1, and shown as mean ± SD of three independent experiments. **B.** Expression of NF-kB in fibroblasts cultured for 24 h in a medium conditioned by DPSCs in the presence or absence of DEX 1 μM. Upper panel: representative confocal microscopy images showing the cytoplasmic and nuclear distribution of NF-kB p65 in the indicated conditions. Lower panel: relative distribution of NF-kB between nucleus and cytoplasm. M: fibroblasts cultured in a non-conditioned medium in the absence of DEX; M+DEX: fibroblasts cultured in a non-conditioned medium, in the presence of DEX; CM NS: fibroblasts cultured in DPSC-conditioned medium; CM S: fibroblasts cultured in a medium conditioned by DPSC stimulated TNF-α and IL-1β for four hours; CM NS+DEX: fibroblasts cultured in DPSC-conditioned medium in the presence of DEX 1 μM; CM S+DEX: fibroblasts cultured in a medium conditioned by DPSC stimulated TNF-α and IL-1β for four hours, in the presence of DEX 1 μM. Scale bar: 20μm. *=p<0.05; **=p<0.01; ***=p<0.001; ****=p<0.0001.

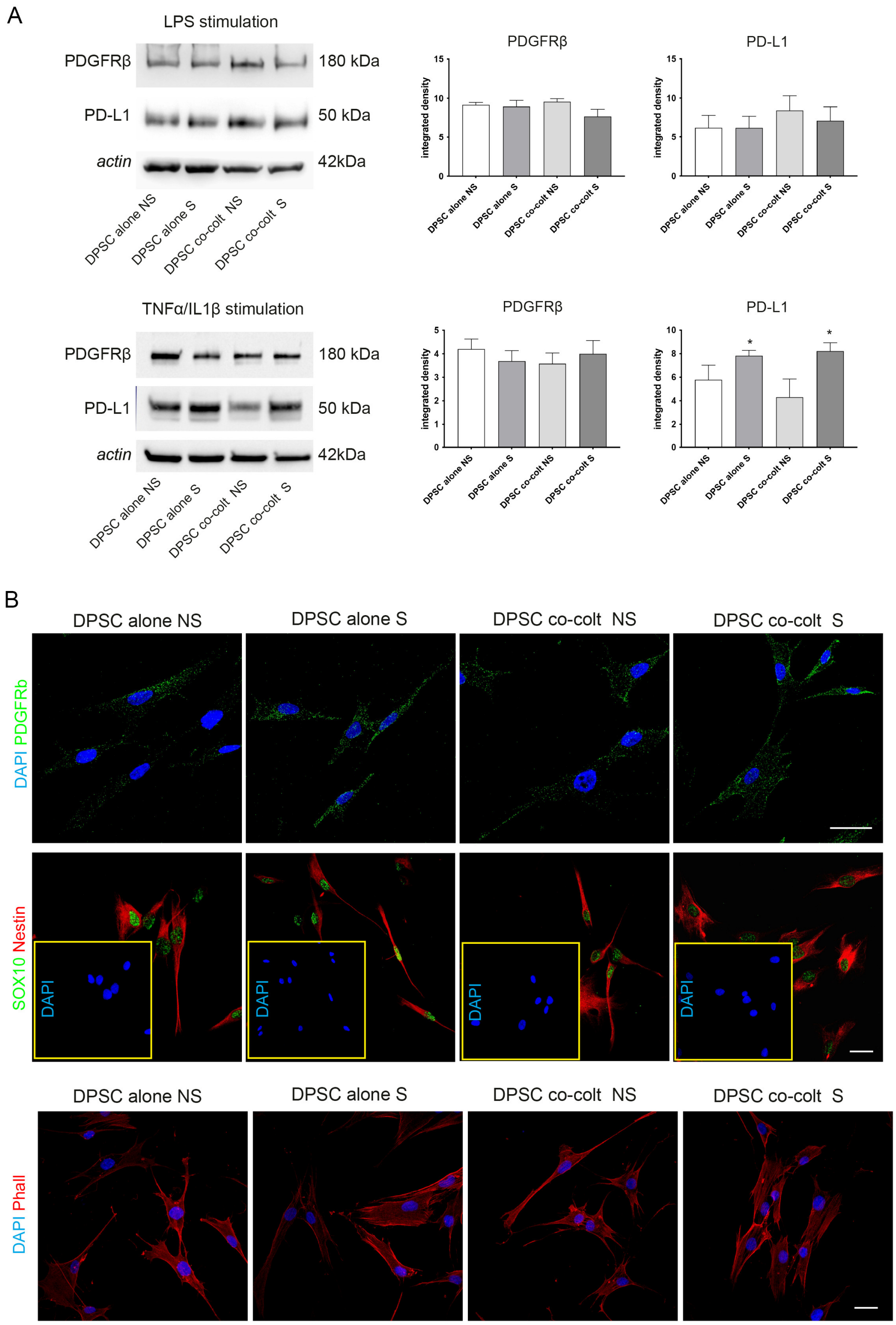

Supplementary Figure S5. A. Representative immunoblots and relative expression of PDGFRβ and PD-L1 in DPSCs treated with LPS (top) or TNF-α+IL-1β (bottom) for 24 hours, alone or in co-culture with fibroblasts. Data are reported as mean ± SD of three independent experiments. *=$p<0.05$. **B.** Representative confocal microscopy images of DPSCs treated with TNF-α+IL-1β for 24 hours, alone or in co-culture with fibroblasts, labeled with anti-PDGFRβ, anti-SOX10, anti-nestin antibodies and AlexaFluor 555 Phalloidin. Nuclei were counterstained with DAPI. NS: non-stimulated, S: stimulated. Scale bar: 20μm.
